# Aristotle’s lobster: the image in the text

**DOI:** 10.1007/s12064-020-00322-6

**Published:** 2020-10-13

**Authors:** Alexander Fürst von Lieven, Marcel Humar, Gerhard Scholtz

**Affiliations:** 1 Institut für Biologie/Zoologie, Freie Universität Berlin, Königin-Luise-Str. 1‒3, 14195 Berlin, Germany; 2 Seminar für Griechische und Lateinische Philologie, Freie Universität Berlin, Habelschwerdter Allee 45, 14195 Berlin, Germany; 3 Institut für Biologie/Vergleichende Zoologie, Humboldt-Universität zu Berlin, Philippstr. 13, 10115 Berlin, Germany

**Keywords:** Aristotle, Animal anatomy, Lobster, Zoological drawings, History of animals

## Abstract

The *Anatomai*, a lost work written by Aristotle, must have contained a collection of various drawings and figures of species as well as their organs. In his texts (mainly the *Historia animalium*), Aristotle is often referring to the drawings after the description of species. Our study applies the method of the comparative view (‘Vergleichendes Sehen’) to provide an access to and reconstruction of Aristotle’s lost illustrations based on his textual descriptions. As an example, we chose the treatment of the European lobster (*Homarus gammarus* L., 1758) in the Aristotelian corpus as a case study. First, we analyse the etymology of the Greek term *astakós* referring to the lobster and provide an overview on the putative synonyms. Second, we confront the textual basis of the description with several questions concerning the degree of abstraction, the relation between text and image, and the spatial orientation of the image. Finally, we present a step-by-step reconstruction of Aristotle’s illustrations of the lobster based on the various passages dealing with its anatomy in the text of the *Historia animalium*. The problems which arise by a confrontation of the textual basis with hypothetical images are discussed at a more general level. We conclude that this kind of a text-based image reconstruction is only possible if the object described by Aristotle is unambiguously identifiable and still visually accessible.

## Introduction: text and image

Language generates images (Nöth 2001:25). As soon as we begin to acquire language, connections are formed between words and the visual image which springs to mind every time we hear or read the word in question. Despite this, texts are limited in their ability to generate visual impressions which actually match reality. When it comes to describing things experienced in a way that is primarily visual, such as spatial relationships and the shape of structures, images are clearer and much easier to understand than texts (Bruhn 2009:16 ff.). For this reason, writings on anatomy, architecture, astronomy, geometry and geography have always been accompanied by images.

In the case of anatomy, the very name of the discipline stems from the title of a collection of images, the *Anatomai*, which formed part of Aristotle’s treatises on zoology.^1^ The only evidence of the existence of these “section drawings” (aná = based on, tomaí = sections) in humankind’s first atlas of anatomy stems from references to figures in Aristotle’s texts themselves and from secondary references to the *Anatomai* in the writings of later authors.^2^ The figures themselves have not been preserved.^3^ The significance of this loss becomes clearest at the points in his works where Aristotle emphasises the parity of text and image:

“For some of these things need to be clarified by an account, others rather by visual inspection.” (*PA* IV 5, 680 a2‒3. Translations of *PA* are from Lennox 2001).

Not only does the conveyance of anatomical information depend on text and images in equal part, the very act of thinking itself is for Aristotle a process of visualisation:

“We cannot think without imagery [*phantásmata*] for the same effect occurs in thinking as is found in the drawing of a diagram […]” (*De Memoria* 449 b31‒450 a2. Translation after Sorabji (1972); slightly modified)

The loss of the figures, then, results in the loss of a critical means of accessing the anatomical content of the zoological treatises. Textually, Aristotle conveys this content by using descriptive phrases, figurative language, metaphors and, in exceptional cases, using new names (e.g., *mýtis* = probably the midgut gland in Cephalopoda, such as octopus, squid and cuttlefish, see *HA* IV 1, 524 b14‒15.). Aristotle was the first person to systematically compare parts of the animal body and thus the first person to have to develop a language to refer to what was previously unnamed (Fürst von Lieven and Humar 2017a). In the *Historia animalium*, he precedes his discussion of animal anatomies with a detailed description of the human body in order to introduce the existent terminology for human body parts and be able to transfer this to animals:

“To begin with, we must take into consideration the parts of Man. For, just as each nation is wont to reckon by that monetary standard with which it is most familiar, so must we do in other matters.” (*HA* I 6, 491 a19‒22. Translation by Thompson 1910)

With this as a starting point, the following questions present themselves:

1. What images do Aristotle’s descriptions of animals convey and how do these images relate to reality (actual animals)?
2. Is it possible to create images based on Aristotle’s texts which visualise his ideas about animal structure and anatomy?
3. If so, what would the relationship be between an image thus generated and the lost images in the *Anatomai*?

There are surprisingly few attempts to reconstruct figures of Aristotle’s anatomical works (e.g., Aubert and Wimmer 1868; Thompson 1910; Peck 1965; Leroi 2014; Fürst von Lieven and Humar 2017b).^4^ However, most of these reconstructions are not based on a transparent and critical discussion of the methodology (see Fürst von Lieven and Humar 2017b). For instance, Leroi (2014) just mentioned that, based on a collaboration with an artist an expert on papyri, Aristotle’s relevant text passages and unspecified examples of drawings from antiquity were used to create the illustrations in his book. By contrast, Fürst von Lieven and Humar (2017b) used the first attempt to reconstruct Aristotle’s image of a sea urchin to provide a detailed step-by-step account on the problems and restrictions to translate descriptions into images. A method that is elaborated and refined in the present manuscript.

## Materials and methods

As a case study, we decided to use the descriptions of the Astakos^5^ (ὁ ἀστακός) in the chapters of the *Historia animalium* and *De partibus animalium* dedicated to crustaceans (*HA* book IV, chapter 2; *PA* book IV, chapter 8). Additional details pertaining to the interpretation of specific structures can be found in *De respiratione* (chapter 18, 476 b), while *De incessu animalium* (chapter 4, 705 a31ff.) contains remarks of significance when it comes to establishing the perspective and orientation of potential images. These texts are compared with preserved lobster specimens of the species *Homarus gammarus* from the zoological collections of the Freie Universität and the Humboldt-Universität in Berlin.

There are various possible ways to approach a comparison between the text and the lines and forms of the structures to which the text probably refers (Fürst von Lieven and Humar 2017b). Firstly, however, it is necessary to establish what species Aristotle is describing. Here, the names he gives the animals in his descriptions and the interpretations of these names in later texts can provide important pointers, although ultimately only being able to recognise in a real, empirically accessible animal all the characteristics listed by Aristotle under a particular name can provide convincing proof that the right species has been identified.

The next question is what perspective Aristotle is describing the anatomical structures from. To answer this, the descriptive texts must be trawled for local prepositions and any relational adjectives derived therefrom, and the more general texts for any concepts relating to body axes. Another matter for consideration is the degree of schematisation involved. Were the illustrations intended to paint as true-to-life a picture as possible, to schematically highlight essential structures, or to be a combination of both? Modern anatomical literature presents images both of bodies in their entirety and of isolated structural details. Here too the question is which of these functions Aristotle’s illustrations fulfilled.

**Fig. 1:**
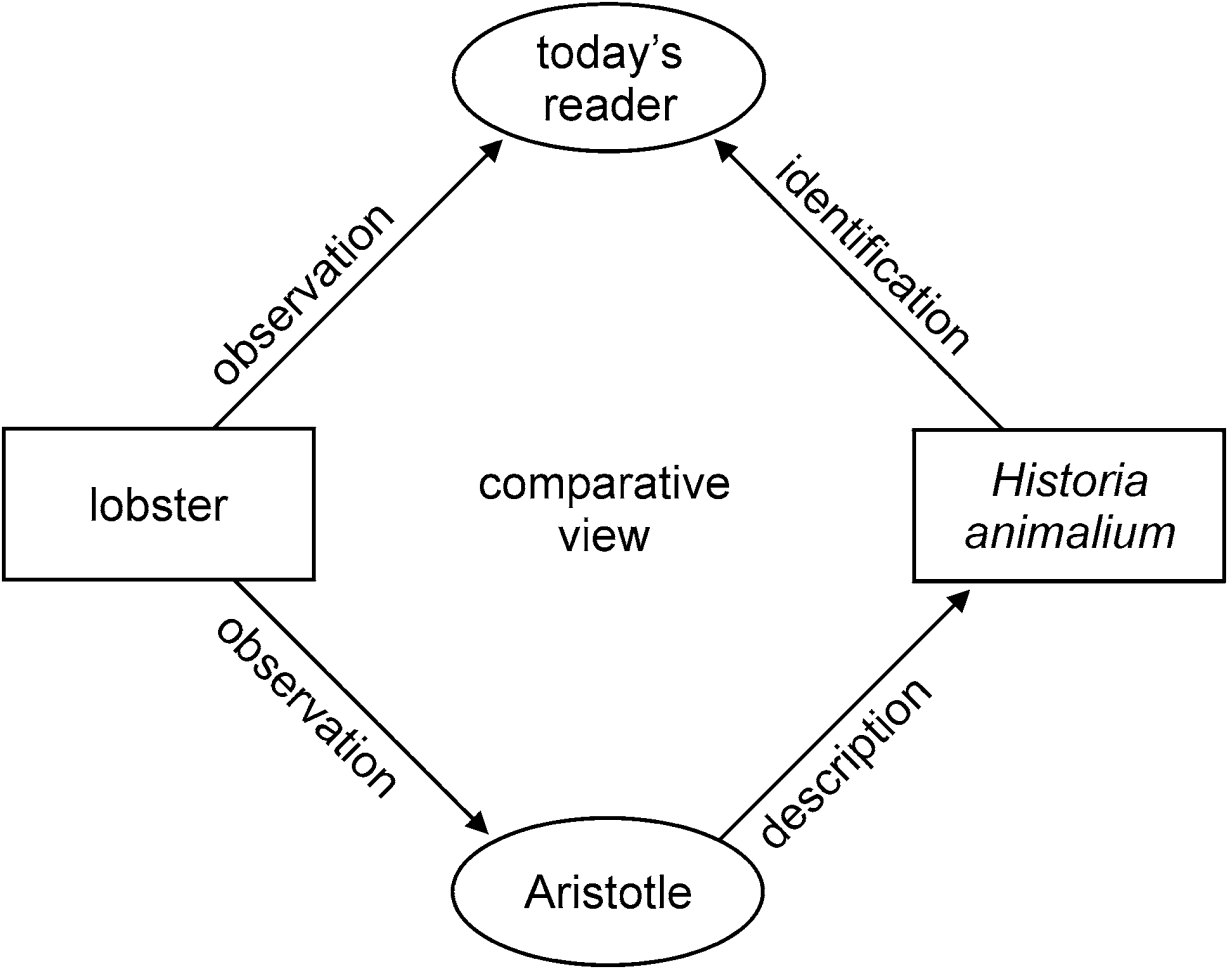
Schematic representation of the method of “comparative seeing” showing the relationship between the textual description and the animal probably described. In order to decide which animal Aristotle was describing, the reader has to compare what is described with a range of possible animal candidates. Identification is made possible by the reader’s own observations

**Fig. 2:**
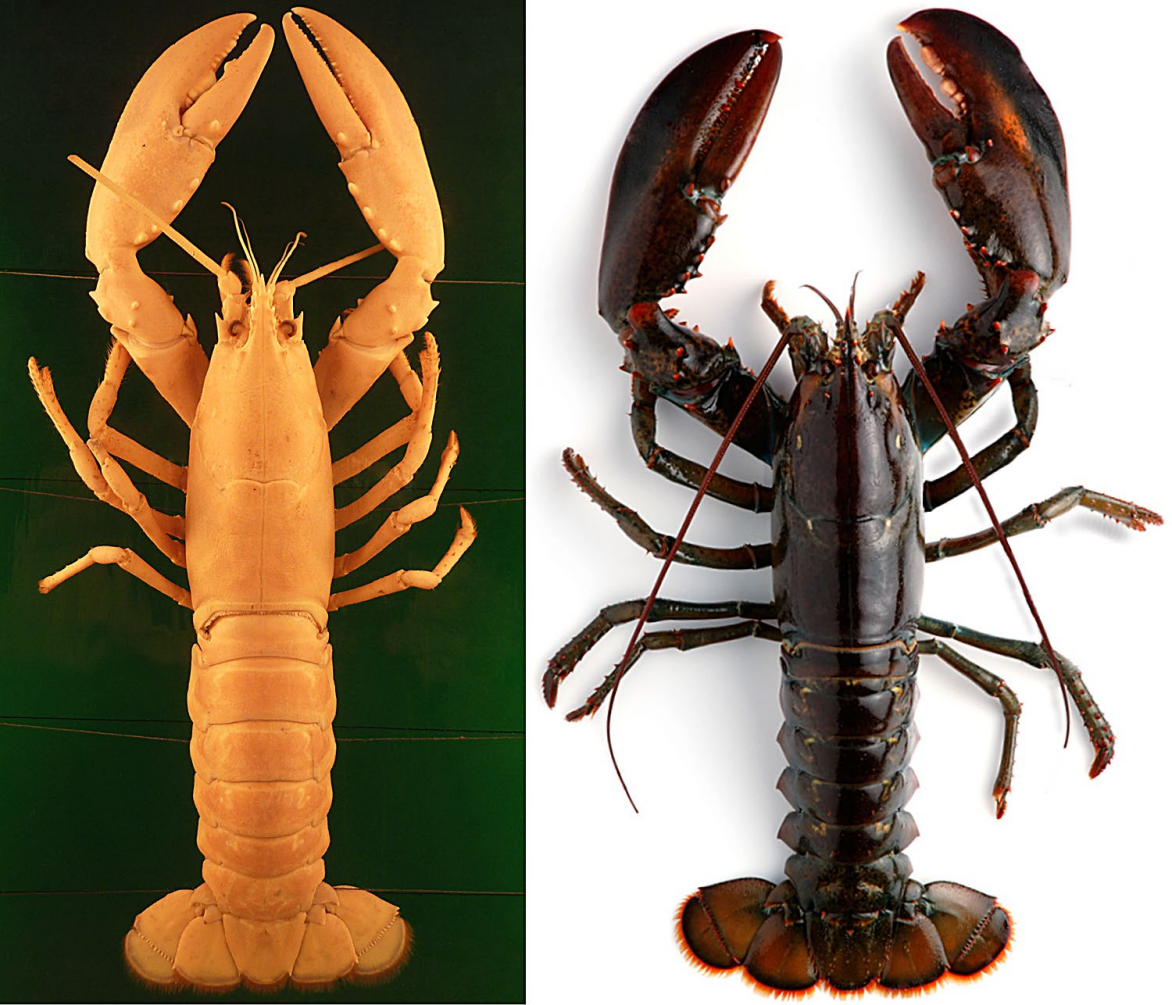
European Lobster specimens. Left: fluid-preserved specimen of a female from the zoological teaching collection at Berlin’s Humboldt University. Although the antennae have been shortened and the colour has faded, the specimen offers an impression of what the lobster on which Aristotle’s description was based would have looked like. Right: photograph of a live male lobster. (adapted from an image published by the Danish Ministry of Environment and Food), the sex evident from the fact that the pleon is narrower than the carapace. The animal displays the dark colouring, which caused Classical authors to refer to it as an elephant and bears its crusher claw on the right

Of equal importance to answering these questions is identifying the structures referred to in the descriptions of the animals. There is a hierarchical relationship between identifying the structures and identifying the species, because the more closely the taxonomical group denoted by an Aristotelian animal name can be narrowed down, the more specific we can be about the appearance of its characters. If we know what structure is being described from what perspective, this can be transferred, with the help of actual animals as models (here preserved lobster specimens), into a drawing. The method is thus a form of “comparative seeing” which reflects the multi-dimensional relationships between the real object, observation, representation and description in the text as it has come down to us (Figs. 1, 2).

**Fig. 3:**
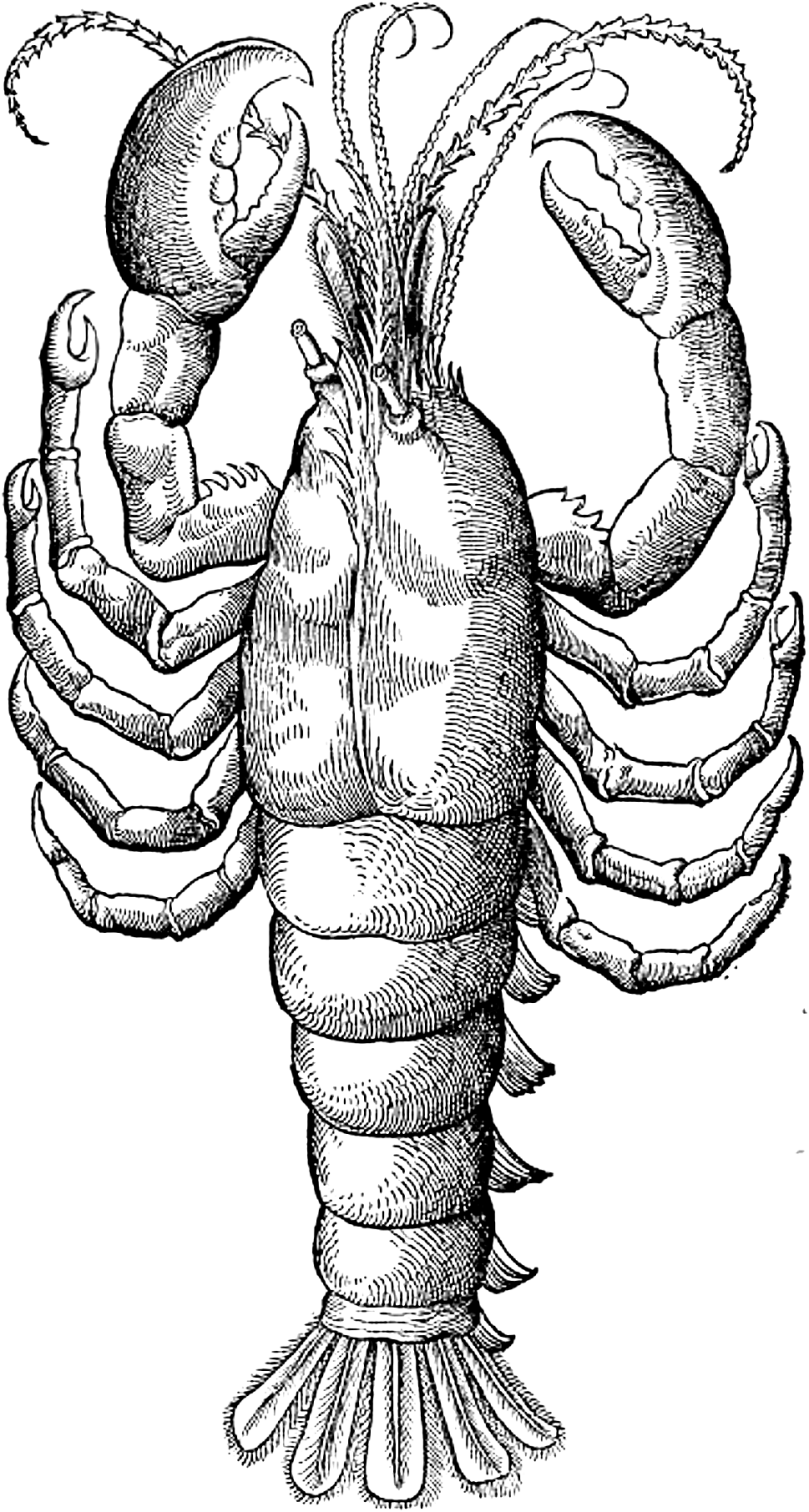
Example of an early illustration of a lobster (from Gessner 1606). Many of the structures listed by Aristotle are clearly recognisable. Least true-to-life are the eyes, antennae and tail fan (see Fig. 2). Interestingly, the way the antennae are depicted as having rings goes back to Aristotle’s reference to “horns” (kérata). The accentuation of the rings is clearly reminiscent of ram or ibex horns. Another striking feature of the illustration is that the pleon is depicted not from a dorsal perspective like the rest of the animal, but from a semi-lateral perspective—potentially in partial correspondence with Aristotle’s description (if his mention of four pleopods is taken as being the view from a lateral perspective; see text)

A further methodological approach to reconstructing the lost illustrations is offered by the astonishingly rich tradition of crustacean depiction (Charmantier 2014). Along with gastropods (snails) and bivalves, crustaceans have always been the most frequently depicted of the marine invertebrates, and impressive forms in particular, such as lobsters, spiny lobsters, crayfish and large crabs, which also serve as food for humans, feature prominently in all kinds of visual depictions, objets d’art and scientific illustrations and have done throughout history. These works document not just the perspective of the individual artists who created them, but the view of crustaceans which prevailed at the time of their production, thus shedding light on lines of tradition in crustacean depiction and departures therefrom (Fig. 3).

For the purposes of our attempt to reconstruct Aristotle’s missing illustrations, the outline of a lobster (see Fig. 2) was reproduced graphically and combined in semi-schematic fashion with the details mentioned in the text. With the exception of the asymmetrical claws, the right and left body sides were mirrored down the vertical axis. The outline is consciously presented here in the draft view of the vector graphic program (Corel Draw 8.0) in which it was created.

## Results of the analysis

### What species is Aristotle’s Astakos?

#### Identifying the Astakos on the basis of Aristotle’s description

In his texts, Aristotle mentions a series of crustaceans to which he refers by different names, characterising them on the basis of where they occur, their anatomical structure or their behaviour. However, his characterisations are heterogeneous to say the least, making it impossible to identify all the species he describes with the same degree of certainty. Some characterisations are so vague as to make identification impossible, while others are surprisingly precise. However, the precision of a description is just one factor in being able to identify a species. The presence or absence of closely related species or similar forms in the same region also play a crucial role. Species which are unmistakeable can usually be identified on the basis of just a few typical characters, or on the basis of specific combinations of characters which only occur in one species.

When describing the Astakos, Aristotle uses the spiny lobsters (Káraboi) as a reference, consistently comparing the structures found in the Astakos with those in spiny lobsters and emphasising similarities and differences between the two. Clearly, Aristotle was able to assume that his readers would be more familiar with the appearance of spiny lobsters than they were with that of the Astakos. This implies that spiny lobsters were then significantly more common in the Eastern Mediterranean than lobsters were.^6^

Aristotle’s description of the crustacean which he terms Astakos makes it possible to identify the species unambiguously as the European lobster *Homarus gammarus* (Linnaeus, 1758) (see also Cuvier 1803; Schneider 1807; Voultsiadou and Vafidis 2007). This certainty results partly from the sheer number of characters Aristotle mentions, which include the long pleon (= abdomen), characteristic spines, the smooth surfaces of the body and details of brood care, and partly from the fact that only one species in the Mediterranean corresponds to the description in the first place. Aristotle describes one pair of large claws and four smaller leg pairs, two of which also bear claws, and this constellation alone limits the possible candidates to a species of lobster or crayfish (Scholtz and Richter 1995). Three pairs of claw-bearing walking legs are only otherwise found in penaeid shrimps and Stenopidea, and in penaeid shrimps all the claws are very small while in Stenopidea those on the third pair of legs are significantly enlarged. All other Decapoda possess either fewer claw-bearing walking legs or none at all. Only two lobster and three crayfish species occur in the Eastern Mediterranean region, and this narrows down the number of species that could be termed Astakos even further.

Aristotle also mentions that the large claws on the first pair of walking legs differ significantly between left and right (a phenomenon we today term heterochely), and this is ultimately the decisive character in enabling us to identify the Astakos as the European lobster. Other heterochelous crustaceans either do not have the combination of characters listed above or do not occur in the Mediterranean. In his description, Aristotle tellingly uses the same diagnostic characters that are still used in field guides today.^7^ Heterogenous claws are sometimes seen in crayfish and lobsters of other species when a claw has been lost and is in the process of being regenerated (the new claw only reaches the original size after several moults, if at all). However, the description of the form and dentition of the claws in the Aristotelian Astakos is so detailed and unambiguous that ultimately only the “crushing” and “grasping” claws of *Homarus gammarus* can be meant (Fig. 2).

At one point in the *Historia animalium*, Aristotle mentions a species of hermit crab which resemble “those little Astakoi that are found in rivers” (*HA* IV 4, 530 a28: […] ἀστακοῖς τοῖς μικροῖς, οἳ γίγνονται καὶ ἐν τοῖς ποταμοῖς·[…]). Adding to an established name an epithet indicating a divergent lifestyle was a common way in Antiquity of creating names for other species, as in the case of *híppos* (horse) and *híppos potámios* (hippopotamus); see Bodson (2005). In the case of the *astakoîs toîs mikroîs, oì gígnontai kaì en toîs potamioîs* Aristotle seems, in pointing out its smallness and its freshwater habitat, to be underlining what is different about the crayfish, which in the vernacular was perhaps also called Astakos like the lobster.

#### Etymology and classical synonyms for Astakos

The way in which the names Aristotle attributed to the species described in the *Historia animalium* have been received over time and the commentaries of later writers on these names ought ideally also to be taken into account in the identification process. In the case of a name as old as astakós, tracing the relationship between it and the species to which it is used to refer is a complex undertaking, made more difficult by the multiple synonyms also in use.

The name astakós is derived via vowel assimilation from ostakós, which in turn is derived etymologically from the syllable ost-, which indicates hardness^8^: astakós can thus be translated as “the hard one”. Clearly, the hardness of the lobster shell was the defining factor in its name.^9^

Alongside the name *astakós*, with its implicit description of the lobster shell, we find the same creature referred to by the names of larger, more commonly known animals^10^ as elephantus (elefant) by Pliny and léon thaláttios (sea-lion)^11^ by Aelian. An extended discussion of the synonyms in antiquity can be found in the original German version of this text (Fürst von Lieven et al. 2017).

#### Modern synonyms for and the taxonomy of the Astakos

While Aristotle uses the word Astakos to refer to the lobster, and in one case the crayfish (see above), the Latinized form *Astacus* is nowadays the genus name for several European crayfish species, including the noble crayfish *Astacus astacus* (Linnaeus, 1758).^12^

The binominal system of species classification was introduced by Carl von Linné (Carolus Linnaeus) (1707‒1778) in the tenth edition of *Systema Naturae* (1758)—that on which today’s zoological and botanical nomenclature is based.^13^ Prior to this, animal and plant names were allocated fairly randomly. Crustaceans were referred to in general as *cancri* or by a local vernacular term. *Astacus* was used by Gessner, for example, to denote the crayfish (*Astacus fluviatilis*) and the lobster (*Astacus*), which he also calls the “großer Meerkrebs” (great sea crab).^14^ At the same time, other authors of the period were using the name *Gammarus* to talk about crayfish and lobsters (von Berniz 1671b). This is probably derived from *cammarus*, a term used somewhat unspecifically for crustaceans in Pliny’s *Historia naturalis* and one which is found in many Early Modern writings.^15^

Von Linné (1758) placed lobsters and crayfish along with all other large crustaceans in the genus *Cancer*, terming the lobster *Cancer gammarus* and the crayfish *Cancer astacus*. Subsequently, however, the differences between the crustacean groups came to be perceived to be so significant that the genus name *Cancer* stopped being used universally and was restricted to just a few species of crab. Johan Cristian Fabricius (1745‒1808) introduced the genus name *Astacus* for crayfish and lobsters in 1775 (De Grave et al. 2009:20), and in 1795, Friedrich Weber (1781‒1823) christened the lobster *Homarus* (De Grave et al. 2009:20). In accordance with the rules of nomenclature that have been in place since von Linné and which since the publication of the tenth edition of *Systema naturae* (1758) have given priority to the first description of a species, von Linné’s species epithet *gammarus* and Weber’s *Homarus*, elevated to the status of genus name, continue to denote the lobster to this day: *Homarus gammarus*.

### What perspective was Aristotle describing the lobster from?

The biggest problem in trying to reconstruct the outline of a lost drawing whose content is now only reflected in an associated text is the question of the perspective from which the author depicted the object of observation. Was he looking at it from above, from below or from the side? The natural position of an animal can provide some idea: one would hardly try to describe a flatfish from a dorsal perspective. In cases where various perspectives are imaginable, a precise analysis of the way the description is formulated can help, as shown by the following example from *Historia animalium*:

With the viviparous quadrupeds the front legs bend forwards and the hind ones backwards, and the concavities of the two pairs of limbs thus face one another. (*HA* II 1, 498 a5‒8. Translation adapted from Thompson 1910)

The “viviparous quadrupeds” are our four-legged placental mammals (excluding bats and humans). Because, unlike in “oviparous quadrupeds” (frogs, lizards, tortoises, turtles and crocodiles), their leg joints are only visible from the side, not from above, it would hardly be logical to illustrate this statement from anything other than a lateral perspective. To establish whether the animal is being described from the left side or the right, the statements in the description can be projected onto the imagined image from left to right in the sequence in which they appear. Looking from left to right along the left side of a four-legged mammal would mean that the front legs came first, as in the text, followed by the hind legs. The animal in this case is therefore facing left.

**Fig. 4:**
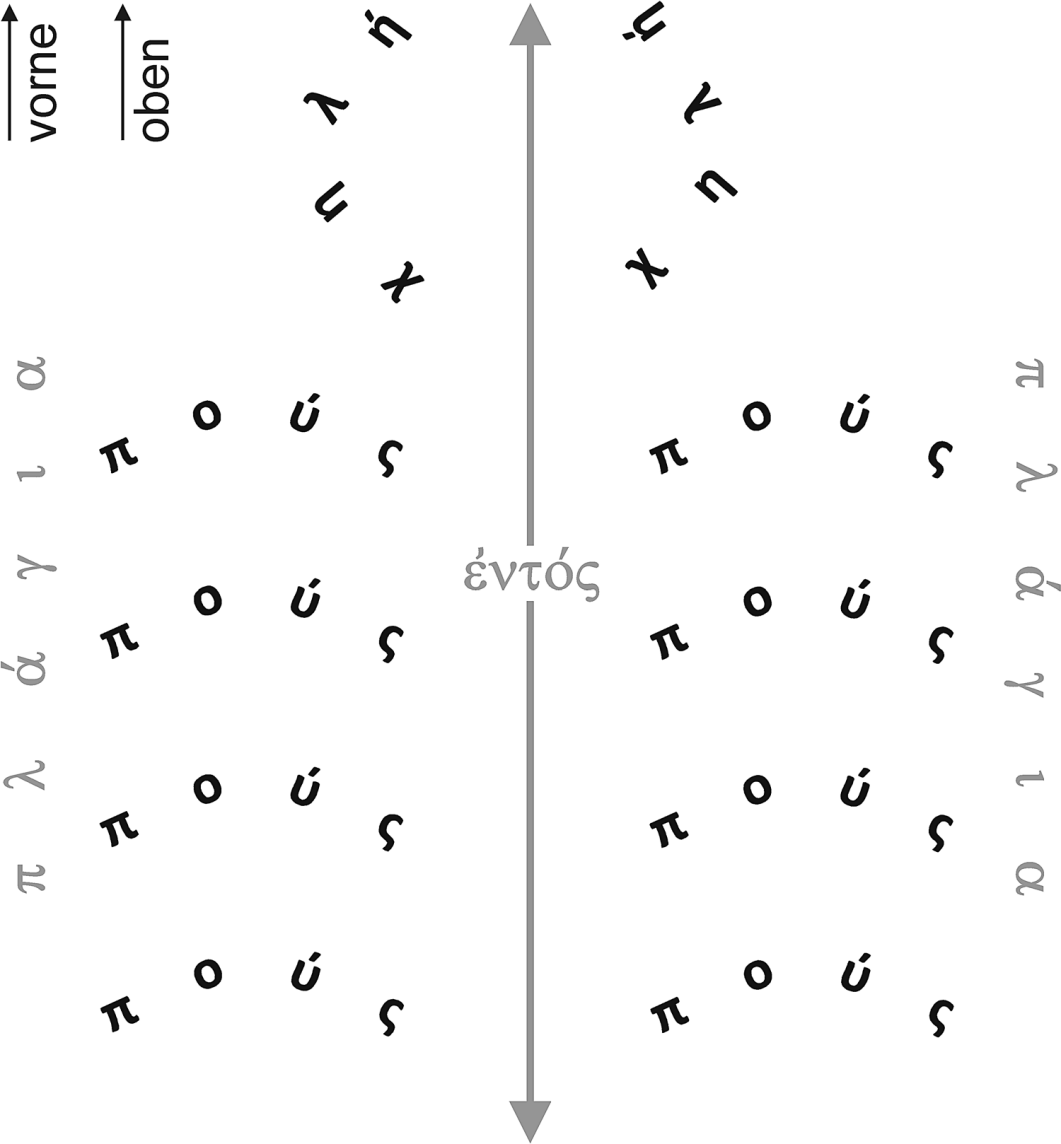
Aristotle’s take on crustacean limb bends. Starting from the description in *HA* IV 2, 525 b24, the subjects (pódes, chelaí) of the predicate “bend” are set out in accordance with the adverbial stipulations (plágion, entós) to visualise what the sentence expresses verbally

The same method can be applied to the description of the crustaceans (Fig. 4):

The feet [πόδες] of all these species [i.e. crustaceans] bend out sideward [πλάγιον] as in insects, the claws [χηλαί], where present, bend inwards [ἐντός]. (*HA* IV 2, 525 b24‒26. Translation slightly modified by us)

When one thinks of a lobster and tries to imagine an illustration showing the claws bent inwards and the legs out sideways, the only two possibilities are that it was drawn from a dorsal or a ventral perspective. As the dorsal perspective corresponds to the way we observe the animal’s natural position, it is likely that the illustration to the text in question was from a dorsal perspective.

Figure 4 arranges the subjects (pódes, chelaí) of the predicate “bend” according to the adverbial stipulations (plágion, entós) in the sentence quoted above to show their hypothesised spatial relationships. The leg positions thus obtained do not correspond exactly to the natural position of the legs in lobsters, as usually only the hindmost leg pairs bend like this (Fig. 2).

**Fig. 5:**
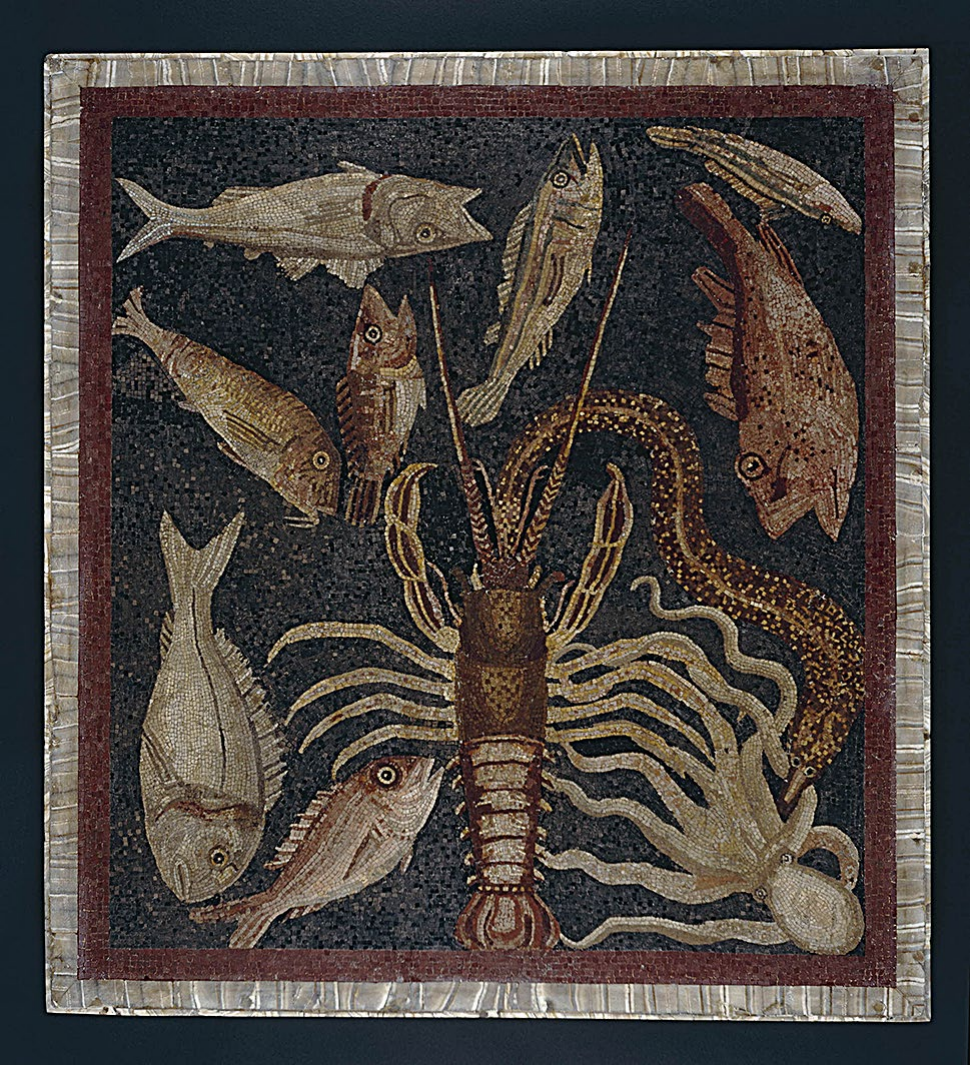
Floor mosaic of marine animals, 1st century AD, Populonia (now the province of Livorno, Italy), London, The British Museum. Reproduced with the kind permission of the ©Trustees of the British Museum

**Fig. 6:**
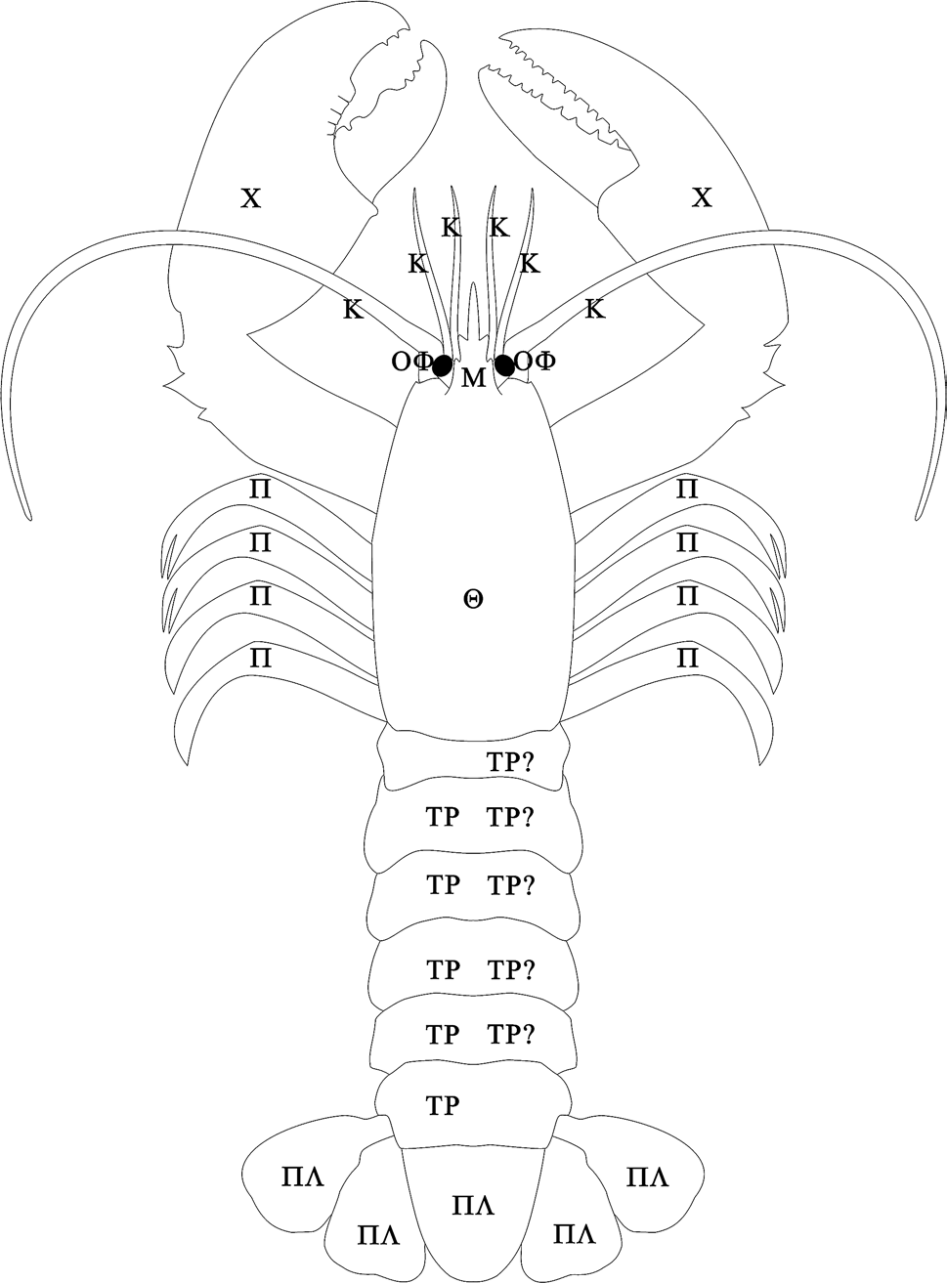
Visual hypothesis of the way the lobster was depicted in Aristotle’s *Anatomai*. When it comes to identifying the five pleon segments (TP) Aristotle mentions, there are two possibilities: either he failed to notice the first pleon segment, which is shortened and partially hidden by the posterior edge of the carapax (labelling scheme on the left side of the pleon), or he counted the sixth pleon segment as being part of the “broad end” (labelling scheme, with question marks, on the right side of the pleon). Upper side (dorsal) perspective. Κ = kéras (κέρας; horn), Μ = métopon (μέτωπον; forehead), Χ = chelé (χηλή; cloven hoof), ΟΦ = ophthalmós (ὀφθαλμός; eye), Π = poús (πούς; foot), ΠΛ = pláks (πλάξ; flat, broad), Θ = thórax (θώραξ; carapace), ΤΡ = tráchelos (τράχηλος; neck)

The front end of the body in Figs. 4, 5 and 6 points upward, as is the case in the vast majority of anatomical drawings of crustaceans today. The question is whether the Ancient Greeks would also have drawn a crustacean with its front end pointing upwards.

What Aristotle regarded as up/top and down/bottom is defined in *De incessu animalium*, where he writes that up/the top [ἄνω] is: “[…] the part from which is derived the distribution of nutriment and the growth […]” and down/the bottom [κάτω]: “the part to which the growth extends and in which it finally ends is the inferior” (*IA* 4, 705 a31ff.; translation based on Forster 1937).

This suggests that he is proceeding from the position of the mouth (up) and the anus (down) in humans. Forward is the direction from which input to the main sensory organs such as the eyes or nose comes, and backward is the opposite direction. Aristotle concludes from this, the first discussion of body axes in animals (Carbone 2016), that while in bipedal humans the directions up and forward are at right angles to each other, in quadrupeds and polypods such as crustaceans they are one and the same (see Figs. 4, 5, 6).

As the right and left sides of the page are naturally associated with the right and left sides of the body of an animal shown from a dorsal perspective, crustaceans (with the notable exception of shrimps) are not going to be drawn facing left as a quadruped shown from the side would. In modern textbook illustrations, the top end of the crustacean is associated with the top edge of the page. It can hardly be argued that the orientation used in today’s textbooks stems from Aristotelian figures that have been unseen for 2100 years,^16^ but it is possible that the anthropocentric definitions of up, down, backwards and forwards used by Aristotle constitute a general principle of human perception.

Amongst the Classical depictions of crustaceans upon which such hypotheses must be tested is a first-century AD floor mosaic of marine crustaceans from Populonia, in what is today the province of Livorno, Italy. This mosaic (Fig. 5) has no clear “up” or “down”. Any of the edges could be the bottom edge, depending on which side the observer is looking at it from. If we take the side featuring the spiny lobster as the bottom, the crustacean is indeed depicted with its front end pointing upwards. The motif depicted in the mosaic—a scene which also features in the *Historia animalium*—does have the spiny lobster in a central position,

For the octopuses [πολύποδες] overcome the spiny lobsters [καράβους], […]. The spiny lobsters overcome the conger eels [γόγγρους], […]. But the conger eels eat the octopuses […]. (*HA* VIII 2, 590 b14‒18)

According to Aubert and Wimmer (1868), however, this passage may be “corrupt” and actually stem from later commentators. Apparently, the same alleged food chain is captured in a saying still popular amongst Italian fishermen: “Il polpo mangia l’aragosta, l’aragosta mangia la murena, la murena mangia il polpo.” (See Bertacchi 1963:71)

### Applying the descriptive terms used by Aristotle to the lobster body

**Fig. 7:**
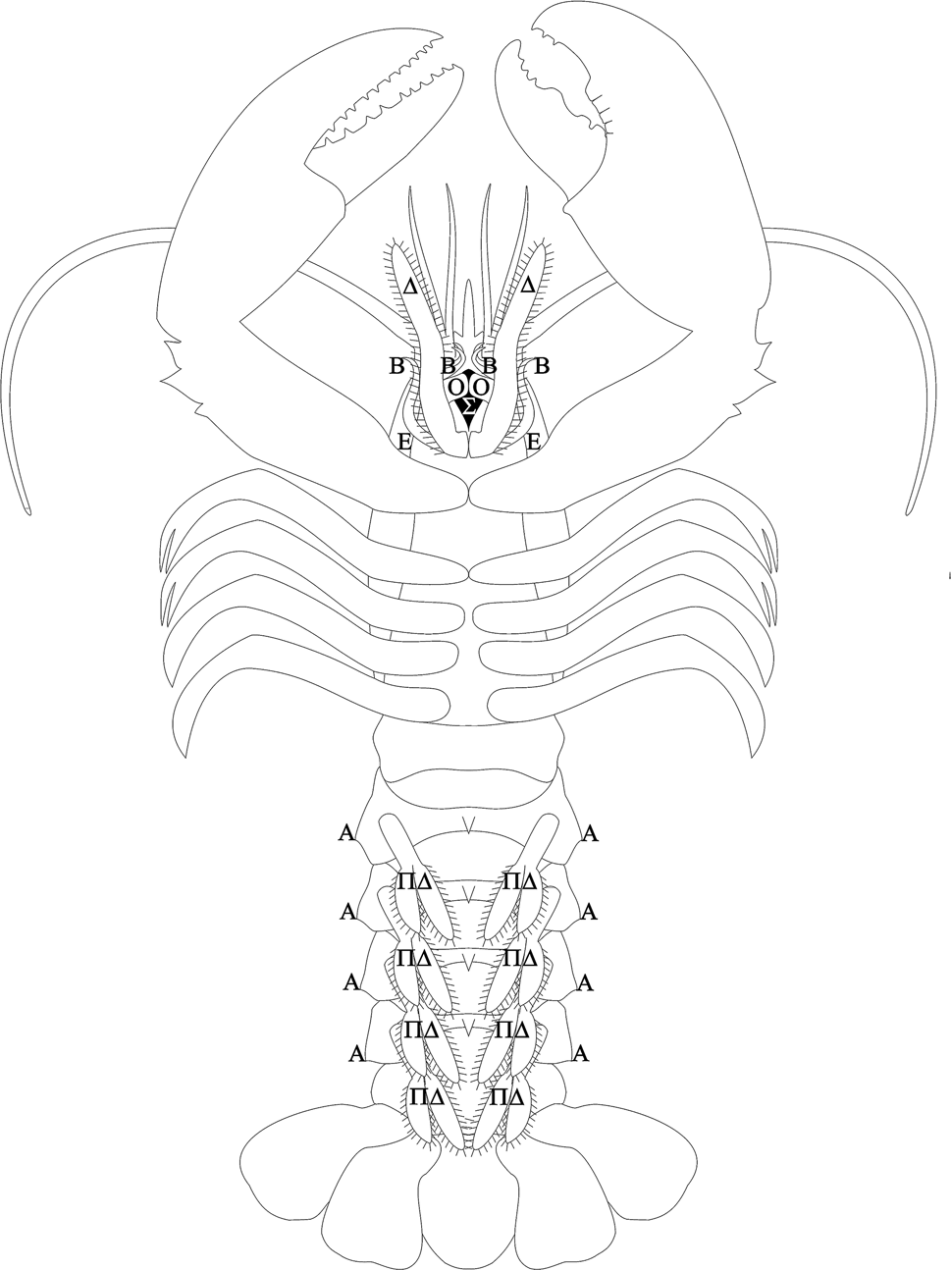
Visual hypothesis of the way the lobster was depicted/represented in Aristotle’s *Anatomai*. Underside (ventral) perspective. Α = ákanthos (ἄκανθος; thorn), Β = branchiódes (βραγχιώδης; gill-like), Δ = dasús (δασύς; hairy), Ε = epíptygma (ἐπίπτυγμα; folded over), Ο = odoús (ὀδούς; tooth), ΠΔ = plakodésteros (πλακωδέστερος; flap-like), Σ = stóma (στόμα; mouth)

In this section, the full description of the Astakos in Aristotle’s *Historia animalium* (book IV, chapter 2) is applied to the structures of the *Hommarus gammarus* body. The results are shown in Figs. 6 and 7, which in terms of orientation reflect the clues about perspective and body axes discussed above. The figures represent two hypotheses, generated by combining statements from all the texts used, of what the drawings of the lobster in the *Anatomai* may have looked like. The structures we identified are labelled in the figures with the capitalised first letter of the Greek term Aristotle used to refer to them.^17^

The lobster is of a dull whitish colour all over, with black mottling. Its lower feet, before reaching the large ones, are eight in number; (the translation of the *HA* here and in the following passages is drawn from Peck 1970)

After describing the colouring of the dorsal side, Aristotle goes on to describe walking leg pairs 2‒5, which in Fig. 6 are labelled (Π) for *pódes* (πόδες).

then come the really large ones, which are much larger and broader at the tips than those of the spiny lobster. Their structure is irregular.

This refers to walking leg pair 1, which end in claws. The claws are referred to in this section of the *Historia animalium* as the broad tips of the large feet, but in other places as *chelé*^18^ (χηλή = cloven hooves). For this reason, they are labelled (Χ).

In the right one the broad tip is elongated and thin, whereas the left one is thick and rounded.

In fact, the asymmetry of the claws is not always this way round—the broader “crushing claw” is sometimes be found on the left, sometimes on the right (see Fig. 2). Aristotle himself discusses this elsewhere, which makes it reasonable to suppose that this part of the text refers to an actual individual. The following very detailed description of the dentition of the crushing claw supports this assumption:

Each is divided at the tip like a jaw and has teeth above and below; in the right one these teeth are small and saw-like; in the left one those at the tip are saw-like, whereas those inside are molar-shaped; in the under part of it there are four of these close together, in the upper part three with interstices between. In both feet (or claws) the upper part moves, and presses down against the lower part. Both are set as play, like bandy legs, as being naturally designed for seizing and exerting pressure.

The comparison of the claws to a jaw (*siagón*, σιαγών) is an image taken from the anatomy of a mammal skull, which Aristotle assumes his readers will be familiar with. The simile also highlights the functional analogy between jaws and claws. The upper dactyl of the claw, the digitus mobilis, corresponds in its mobility to the lower jaw (mandibula) and presses against the immobile lower dactyl (digitus fixus).

Above the two large ones are two others, covered with hairs, a little below the mouth; and below these are the gill-like parts round the mouth, which are hairy and numerous. The animal keeps these in movement the whole time, and bends the two hairy feet and draws them in towards its mouth. The feet near the mouth also have fine appendages.

For it to be possible to make statements about position relative to the mouth, the lobster must be being viewed from a ventral perspective (Fig. 7). From this perspective, the origin of a further “leg pair” is visible between the origin of the first claw-bearing walking legs and the mouth opening, or as the text puts it “above the two large ones” and “a little below the mouth”. Aristotle is talking here about the inner branches of the third maxillipeds, which are indeed covered with hairs. The mouth is labelled (Σ) for *stóma* (στόμα) and the third maxillipeds (Δ) for *daseîs* (δασεῖς = hairy parts).

The gill-like structures said to be “in movement the whole time” would correspond to the flagella-like outer branches of the maxillipeds. “Below” the 3rd maxillipeds thus means here not towards the bottom of the page but “covered by the 3rd maxillipeds”, i.e. the outer branches of the 1st and 2nd maxillipeds. The “fine appendages” are the outer branches of the third maxillipeds. All these outer branches are labelled (Β) for *branchióde* (βραγχιώδη^19^), “gill-like”.

The mention of “gill-like” structures which are constantly in movement causes the zoologist to ask whether Aristotle also said anything about respiratory water flow. And indeed, two sentences following the description of the lobster in *Historia animalium*, book IV chapter 2 are dedicated to this very subject:

All animals of this sort [i.e. the malakóstraka = crustaceans] take in sea-water by the mouth, and having taken it in the crab discharges it while slightly closing up this part; the spiny lobster discharges it by the gill-like organs […]. (*HA* IV 2, 526 b18‒20. Translation slightly modified)

This statement contradicts what is generally taught in today’s text books about the direction of respiratory water flow in decapod crustaceans, which is that the water taken in during respiration is drawn along the rear lateral edges of the carapace into the gill chambers and leaves the gill chambers next to the mouth below the folded down front edges of the carapace. However, it is also taught that respiratory water flow can be reversed, e.g. in brachyuran crabs, to rinse the gills of any dirt which may have got in (Gruner 1993:943). The citation below from *De respiratione* shows that in this (later) treatise, Aristotle accurately describes the direction of the discharge of water.

However, Aristotle did not interpret the intake of water by crustaceans as respiration. Respiration for him was solely the intake of air, as explained in *DR*, and its function was to provide the cooling required by “warm”, blooded animals. Though fish may be slightly cooler than land mammals, they are still blooded animals, and Aristotle saw a connection in both between the blood vessels and that part of the body into which the external medium is received, in land animals the lungs, in fish the gill pouches or cavities. He thus came to the conclusion that, like the intake of air in blooded land animals, the intake of water in fish was for cooling purposes. Aristotle did not recognise the gill chambers in crustaceans and believed that, as they were bloodless and would more or less take on the temperature of the external medium, they did not need cooling anyway. He saw the intake of water simply as a side-effect of feeding, as explained in *DR* 12, 476 b33‒477 a4:

[F]or each of these species [i.e. the crustaceans] is of low temperature and bloodless, so that they are sufficiently cooled by the surrounding water; but they admit water in feeding and so must expel it so that the water may not flow in as they are absorbing food. The crustacea discharge the water through the folds next to the hairy parts […]. (Translation based on Hett 1936, slightly modified.)

This is an example of a term (“the hairy parts”) having once been found for a structure reappearing in another treatise despite being completely unidentifiable out of context. The “folds” can be identified as the front edges of the carapace as seen from a ventral perspective. They are labelled (E) for *epiptýgmata* (ἐπιπτύγματα = folds).

They ought to be of same size, each on a whole page including captions, and face to face.

The function of these folds, in Aristotle’s eyes, was to discharge the water ingested with food, which is also what he (again inaccurately) thought whales were doing when they spout water from their blowholes (see *DR* 12, 476 b). The description of the lobster continues:

The lobster has two teeth, like the spiny lobster, […]

For Aristotle, the essence of a structure is its purpose (See, for instance, *PA* I 5, 645 b14‒20). We can thus be certain that he does not mean “teeth” in a descriptive sense, as this would be expressly formulated so as to indicate an analogy, as in the description of the claws: “*like* a jaw [with] teeth above and below”. Here he actually means teeth, in the same way as humans have teeth. The analogous mouthpart structures in crustaceans are the mandibles (homonymous with the above mentioned term ‘mandibula’ for the human jaw), which is what Aristotle is referring to here. They are labelled (O) for *odóntes* (ὀδόντες = teeth).

[…] and above them are the large horns [kérata], quite long, but shorter and much finer than those of the spiny lobster; then four more [horns] similar to these in shape, but shorter and finer. (Translation modified)

Examples of horns being used as a metaphor for antennae are plentiful even today—think of “longhorn beetles”. In the case of the lobster, the “two large horns” are the second antennae and the “four others” are the first antennae. They are labelled (K) for *kérata* (κέρατα = horns).

Above these [i.e. kérata] are the eyes, small and insignificant, not large like those of the spiny lobster.

“Above” the horns, which means we have clearly returned here to a dorsal perspective (Fig. 6), are the eyes. Aristotle identifies them intuitively, like any observer would, without actually having tested their function as optical sense organs. They are labelled (OΦ) for *ophthalmoí* (ὀφθαλμοί = eyes).

Over the eyes is a pointed rough projection, like a forehead, larger than in the spiny lobster.

This pointed rough projection can be identified as the rostrum, a spiny beaklike structure which projects between the eyes. It is labelled (Μ) for *métopon* (μέτωπον = forehead).

And in general, the facial part is more pointed, and the breastplate [*thórax*] is much broader than in the spiny lobster. (Translation slightly modified).

“Thorax” (*thórax*) originally meant the breastplate of a Greek soldier’s armour (this is the way in which Homer uses *thórax*; see *Iliad* 11, 347 and 23, 560). Later, as an anatomical term, it came to mean the part of the rump covered by the breastplate. In the case of the lobster the term clearly describes the exoskeleton of the walking leg-bearing section of the body. The front end of the thorax merges with the head region, leading zoologists to talk about a “cephalothorax”. When the head region is subtracted, we are left with the “carapace”, which is what Aristotle probably meant here. We label it (Θ) for *thórax*.

Of the eight feet, four are cleft at the tip and four are not. (Translation slightly modified).

Aristotle is talking here about the second and third pairs of walking legs, the penultimate article of which bears a cone against which the last article can be moved, forming a pincer-like structure. As Aristotle only talks of “cloven” tips, they are drawn as if the last (foot) article was cloven, which it is not in reality.

The region of the ‘neck’ as it is called is divided externally into five portions, and the sixth is the broad region at the end, which has five flaps.

Further remarks in the text make it clear that the structure referred to as the ‘neck’ [*tráchelos*] is the rear section of the body, the tail or pleon.^20^ Indeed, the French for a lobster’s tail continues to be *col*, which means also neck. The word Aristotle uses here, *tráchelos,* means not just the human or mammalian body part “neck”, but any neck-shaped narrowing of a contour, like, for example, a bottleneck. The five segments of the pleon are labelled (ΤΡ) for τράχηλος = neck. The five flaps at the end correspond to the end part of the pleon (telson) and the extremities of the sixth abdominal segment (uropods) which lie to either side of it and whose inner and outer branches broaden out into flap-like structures. The flaps are labelled (ΠΛ) for *plákes* (πλάκες = flaps).

The [flap-like] inner parts, upon which the female lays its eggs, [are] four in number and hairy.

The pleon is dorsally convex and ventrally concave. As the convex side of an arc can be described as the “outer side” and the concave side as the “inner side”, the previous sentence was clearly describing the dorsal side of the lobster, whereas this one, which discusses the hairy [inner] parts “upon which the female lays its eggs”, describes the ventral side (Fig. 7). On the basis of the function attributed to the “hairy parts” they can hardly be anything other than the extremities of pleon segments 2‒5 (the pleopods), whose function as egg carriers Aristotle describes clearly in spiny lobsters (*PA* IV 8, 684 a18; *GA* III 8, 758 a13). However, four pleopods are only visible from a side view, so in correspondence with the previous sentence, to which this one refers and which ends with the description of the “five flaps” of the tailfan, the number 4 could be being used here to describe a series running perpendicular to the longitudinal axis. In this case, it would refer to the branches (inner and outer) of the pleopods of a single segment. As the branches of the pleopods in female lobsters do not broaden out to the extent that they actually overlap, as is the case in female spiny lobsters, both are clearly visible, at least in fresh specimens.

The description here is inconsistent, as previous passages always mention the total (bilateral) number of extremities. The comparison to flaps comes from the passages in the text in which the pleopods of female spiny lobsters are described. The pleopods are labelled (ΠΔ) for *plakodéstera* (πλακωδέστερα = flap-like).

On each of these parts just mentioned there is a short straight spine [thorn] pointing outwards.

No structures which would fit with the formulation “on each of these parts just mentioned”, i.e. the pleopods, are actually to be found on the pleopods themselves. There are two possibilities for what could be meant: firstly, four small spines on the ventral side of the pleonic segments (Fig. 7), and secondly the thorn-like structures (epimeres) in which the lower edges of the dorsal plates of the pleonic segments (tergites) end. These are located parallel to each pleopod and are particularly conspicuous from a lateral perspective. An alternative explanation for Aristotle’s citing four rather than eight pleopods (see above) may be that he was describing a lateral view of the pleopods and spines. This rather vague hypothesis allows us to assume that the spines in question are the pointed ends of the epimeres. The spines are labelled (A) for *ákanthos* (ἄκανθος = spike/barb).

Its body as a whole and particularly the parts round the thorax are smooth, not rough as in the spiny lobster, though on the large feet the outer portion carries larger spines.

Aristotle only mentions the outer spines on the first pair of walking legs, so only these have been drawn. They are unlabelled.

At the end of the description in *Historia animalium* it is noted that the asymmetry of the claws is not fixed in terms of left and right.

No difference can be detected between the male and female: they both have one claw (whichever it may happen to be) larger than the other, and neither sex ever has both claws equal in size.

This phenomenon is discussed in more detail in *PA* IV 8, 684 a26‒684 b1, in the light of the Ancient Greek belief in the superiority of right-handedness.

The spiny lobsters and the crabs all have the right claw larger and stronger; for all animals naturally do more things by means of the parts on the right side; […]. The lobsters alone have one claw or the other, whichever one it chances to be, larger, in both the females and the males. They have claws because they are in the kind that has claws; while they have this part randomly distributed because they are deformed, and do not use it to do what claws are naturally for, but for the sake of locomotion.

It is at the end of this observation that the sentence is found which refers to the existence of drawings of crustaceans in the *Anatomai*.

Each of the parts - what their positions are and what differences there are from one animal to another, including the way in which males differ from females - should be studied with the help of the dissections [*Anatomai*] and the “enquiries into animals”. (*PA* IV 8, 684 b1‒5)

This passage also makes clear that the illustrations in the *Anatomai* can be assumed to have included both drawings of the entire animal and drawings of isolated structural details.

## Discussion

Aristotle’s zoological texts constitute the first systematic attempt to catalogue natural objects descriptively and present them in a way comprehensible to the reader (Fürst von Lieven and Humar 2017b). They are not, essentially, descriptions of images. However, as is the case in modern works on anatomy, the text and images in the zoological treatises are, in parts at least, clearly to be understood as a unit. Verbal and visual documentation take place as parallel and closely intertwined processes. This means that the images contain information which does not appear in the text, and vice versa.

Nevertheless, Aristotle’s texts are treated here as descriptions of images, and used in order to generate images. Our hope is that the images thus created will provide an idea of those which the illustrator of the *Anatomai*, whether it was Aristotle himself or an artist working for him, created of the described object. Justification for this method of proceeding lies in the very term “image” itself. A definition of the term as broad as the one Horst Bredekamp (*1947), harking back to Alberti, suggests in his *Theorie des Bildakts*^21^ enables us to interpret a dead lobster, killed and arranged in some way so as to permit examination, as an image. This being the case, Aristotle’s description of the lobster is, in a way, a description of an image after all. And the fact that the description allows us to identify the precise species of lobster in question provides critical access to the image in question.

An image, which serves to identify a biological object uses abstraction and typification to represent those characters which are deemed to be essential. The same applies to descriptive texts, which also generalise the individual and specific and emphasise that which is characteristic.^22^ In principle, this should make it possible to convert the one method of characterising an object into the other, i.e. to translate an image into text and vice versa. Ekphrasis, the practice of verbally describing something visual as vividly as possible, is a tradition which has existed since Antiquity, an ever-evolving art form in its own right (see, for example, Bruhn 2009:70). The reverse process, turning descriptive texts into images, is practised in history painting, film adaptations of literature, and in the theatre, but hardly plays a role at all in scientific illustrations. The few documented examples show dramatic differences between images produced on the basis of a verbal description and images produced by an illustrator who is actually able to look at the described object (Heller and Reble 1990, Fürst von Lieven and Humar 2017b). The linguistic means by which descriptive texts can generate mental images, i.e. metaphors, analogies and descriptive phrases, are clearly too ambiguous to permit a process that could be called ekphrasis in reverse.

And yet the fact that it is possible to look for species which correspond to Aristotle’s objects of observation puts the modern reader in a position to be able to interpret Aristotle’s zoological texts with a precise knowledge of the object of his descriptions and deliberations. The possibility of direct observation is a general advantage of texts which describe empirically accessible, material objects over texts which deal with constructs such as ethical principles (Fig. 1). While significant problems arise if the socio-cultural context is not taken into account when comparing culturally influenced values or feelings such as shame (see, for example, Harré 1986:12), this is absolutely not so when animals or descriptions of animals are compared. The problem in the first case is that there is no standard understanding of shame across different cultures—the term is a compound of various interculturally divergent social behaviour patterns. In the case of an animal description, however, once the organism in question has been identified with certainty, comparisons can be drawn which refer solely to the object—it is simply not necessary to specify an exact social context.

Aristotle’s description of the Astakos is precise enough to enable us to identify the animal as the European lobster *Homarus gammarus*. In fact, it is so precise that large parts of the description must have referred to a specific individual. This is the only explanation for Aristotle’s placement of the crushing claw on the left and the detailed description of its dentition in the first part of *Historia animalium* (book IV, chapter 2). At the same time, it is reasonable to suppose that the illustration in the *Anatomai* would have been intended to represent all lobsters, in the same way that the text includes many generalising formulations too.

In botanical and zoological illustrations in particular, and the texts which accompany them, the individual always functions as a means to demonstrate something about a larger context, and as the representative of a group of organisms (Bruhn 2009:165). Furthermore, “observations and the notation of observations in nature studies […] should not simply be taken as documentation of that perceived by the senses” but as a reference to “that which is already known” and to prevailing theories in the field in question (Breidbach 2005:63; translation our own). The way this knowledge and these theories manifest themselves differs, however, between written descriptions and illustrations. It is possible in descriptive texts simply to leave out aspects not considered to be essential, whereas an illustration must always be more complete in order to show the structures in question in their proper context. When Aristotle speaks of the lobster’s “split feet”, for example, in order to distinguish it from other crustaceans, he does not need to mention which of the eight walking legs the description applies to unless this is relevant to the distinction. In the drawing, on the other hand, the illustrator has no choice but to accurately depict the position and nature of the split, not to mention the shape of the feet (which are not like those in humans or ungulates, but like those in other crustaceans).

It is clear that Aristotle resorts to abstraction and schematisation in his description of the lobster. The text alone does not permit us to imagine a lifelike lobster—rather, it emphasises those aspects which Aristotle deemed to make up the essence of the animal. In other words, it reflects Aristotle’s theories about animals in general and about crustaceans and the lobster specifically. But the illustration that went along with it is also an abstraction and must inevitably have been shaped by the types of schematisation present in the text (see above).^23^ In Fig. 7, for example, all the mouthparts not mentioned by Aristotle have been omitted, a simplification that is also reasonable to assume for the relevant original illustration in the *Anatomai*. On the other hand, Fig. 6, which is from a dorsal perspective, does not show the third maxillipeds, though they certainly would have been visible from above. But because they are not mentioned in the description of the upper side of the lobster, they are omitted here, too. The schematisation displayed by Figs. 6 and 7 is partly the result of such omissions and partly the result of the impossibility of hypothesising the exact course of the lines that might have made up the illustrations in the *Anatomai*. A compromise had to be found between the outline of the model, a real lobster, and a simplification of this outline that would permit identification without implying that the original illustrator tended either towards exaggerated naturalism or towards excessive schematisation, for there is no grounds to suggest either of these extremes.

Figures 6 and 7, which were produced by visualising, using a real lobster as a guide, the statements made in the texts, contain everything which, according to the texts at least, Aristotle must have seen on the lobster, and leave out all structures he does not mention. Their being labelled with the original Greek first letter of the descriptive expressions used creates an additional bridge back to the text. The pictures show at a glance how precisely Aristotle examined the lobster. In this they achieve significantly more than the text, which, via metaphors, analogies and descriptive phrases can only hint at the forms he saw, e.g. by comparing the digiti (digitus fixus and digitus mobilis) on the claws with a jaw, or the protuberances on their inside edges with teeth. The only way any text could be more specific about shape would be by listing coordinates round the outline of the structures in question.

The picture’s informational bonus comes from the real-life model, though the model is also, in some way, present in the text thanks to the fact that it is possible to identify the animal being described. It would be nonsense to depict the *chelé* described in the text as unlike the claws of a lobster just because their exact outline is not evident from the text. As such, it is possible even with all due caution to take the results of our reconstruction attempt as a feasible visual rendering of Aristotle’s notion of a lobster. Figures 6 and 7 provide a well-founded indication of what the pictures of the lobster in the *Anatomai* must have looked like. As the lost images cannot actually be brought back via this pictorial reaction to the text, the figures have consciously been left as the ultrathin outlines generated by the draft mode of the graphic program—a decision which reveals the schematic character of the reconstruction.

The figures can also be understood as a new way of commenting on Aristotle’s zoological texts. One of the tasks of classical philology has always been to comment on Classical sources, and in the case of Aristotle’s zoological treatises, images can provide a new starting point for such commentaries. The requirements of turning an expression or descriptive phrase into an image force new, as yet unposed questions to be asked of a text (e.g. what perspective is the author describing from, which at the same time is a question of the author’s underlying conception of that which is being described).^24^ Questions such as these involve more than confronting, or rather identifying, Aristotle with current knowledge by translating his words into modern zoological terms. They involve an attempt to create hypotheses about how Aristotle saw and thought of his objects of investigation. They lead back to the earliest origins of scientific zoology and make “comparative seeing” possible over millennia.

## Footnotes

1 Aristotle’s works on biology include the ten-book treatise *Historia animalium*, the treatise *De partibus animalium*, which looks at animal organs and provides explanations for their origins, the shorter treatises *De motu animalium* and *De inscessu animalium*, which are about animal movement, and a treatise on animal reproduction, *De generatione animalium*. The titles of these works are traditionally cited in Latin and will be abbreviated here as follows: *Historia animalium* (History of animals) = *HA*; *De partibus animalium* (Parts of animals) = *PA*; *De motu animalium* (Movement of animals) = *MA*; *De incessu animalium* (Progression of animals) = *IA*; *De generatione animalium* (Generation of animals) = *GA*; *De respiratione* (On respiration) = *DR*.

2 The Roman author Apuleius (2nd century BC), for example, mentions this work in his *Apologia* (36, 11‒20 and 40, 14‒17).

3 Although this anatomical atlas has not survived, the *Anatomai* have been the object of various recent philological and historical investigations; see Stückelberger (1993, 1994) and Hellmann (2004). A current overview can be found in Fürst von Lieven and Humar (2017b:74‒75).

4 For a discussion of Aristotle’s use of diagrams and tables see Natali (2013: 113 ff.).

5 In the following, the term Astakos will be capitalised when it is used to refer to the lobster as a biological species and appear in lowercase and with an accent (as a transcription of the Greek) when used to refer to the word as such.

6 A random sample of Classical representations of crustaceans appears to confirm this impression—spiny lobsters are found with the greatest frequency by far. See Charmantier (2014).

7 In Erwin Stresemann’s (1889‒1972) widely used field guide to the fauna of Germany (Exkursionsfauna von Deutschland), the entry for Astacidea (freshwater crayfish and lobsters) reads: “Elongate body dorsoventrally flattened. Pleon [abdomen—the authors] robust with clear epimeres [lateral plates - the authors] and large tail fan. […] The first three walking legs bear claws, the first walking leg is by far the most sizable”. Our translation. The first character used to distinguish *Homarus gammarus* is “Clear disparity between claws on first walking leg: bulky crushing claw and more slender grasping claw”. Hannemann et al. (1992)

8 See Gessner (1606). The same phenomenon is observed in the terms ὀστέον (ostéon, bone) and ὄστρακον (óstrakon, shard of pot and mollusc shell); for the etymology see Frisk (1960:167).

9 The hardness of the carapace is mentioned by other Greek authors; see Oppian *Halieutica* 1, 259‒261.

10 Much in the same way that certain moths are referred to in German as “owls” or “bears”.

11 Probably a squat lobster, see Fürst von Lieven et al. (2017).

12 It remains unclear which species of crayfish occurring in the Eastern Mediterranean region Aristotle meant.

13 The first part of botanical and zoological binomina identifies the genus to which the species belongs, and the second the species itself, e.g. *Homo sapiens* for humans. See von Linné (1758).

14 Gessner (1670). In the Latin edition from 1606, however, Gessner refers to the lobster as *Astacus marinus*. Gessner (1606), see also von Berniz (1671a).

15 See Koutrakis et al. (2009), according to whom Theodorus Gaza (1389‒1475) rendered Astakos as *Gammarus* in his translation of Aristotle’s works into Latin, after which crayfish and lobster were referred to in scientific treatises as *Astacus* and in more popular texts as *Gammarus* during the early modern period. This distinction does not appear particularly plausible, however.

16 One of the last people to encounter them may have been Apuleius; see footnote 2.

17 This method of labelling is based on what is known of the labelling practice in the *HA*. There are two passages which show that capital letters were used to label the figures, one being the description of the illustration showing the male genitalia in *HA* III 1, 510 a29‒32: “All these descriptive particulars may be regarded in the light of the accompanying drawing, wherein the letter A marks the starting point of the ducts that extend from the aorta; the letters KK mark the heads of the testicles and the ducts descending thereunto; the ducts extending from these along the testicles are marked ΩΩ; the ducts turning back, in which is the white fluid, are marked BB; the penis Δ; the bladder E; and the testicles ΨΨ” (Translation adapted from Thompson 1910). The second passage is in *HA* V 18, 550 a25‒26. See Fürst von Lieven and Humar (2017b:76), for a more detailed discussion.

18 See, for instance, *HA* IV 2, 527 b5 and *PA* IV 8, 684 a27.

19 More rarely, the word βραγχιοειδῆ is used (also translatable as “gill-like”); see *HA* IV 2, 526 b20. The word βραγχοειδῆ is also found once in *PA* IV 8, 684 a21.

20 Aristotle uses various terms for the crustacean pleon, including *kérkos* (κέρκος, literally “tail”) in *PA* IV 8, 684 a15 and *ourá* (οὐρά, e.g. in *PA* IV 8, 684 a3). At this particular point in his description of the lobster he indicates he is using a metaphor (“the ‘neck’ as it is called”). There is a need for an in-depth analysis of the semantic field used to describe these structures, and for research to shed light on whether the various terms are used synonymously or in relation to specific species.

21 Bredekamp (2010:35) remarks: „[…], dass von Bildern zu sprechen ist, sowie Naturdinge ein Minimum an Spuren menschlicher Bearbeitung aufweisen.“.

22 As Bredekamp (2010:39) puts it: „Worte wie Gemälde besitzen durch Ihre Fähigkeit zur verknappenden Darstellung vielmehr jene klassifikatorische Kraft der bezeichnenden Wiedergabe.“.

23 Heiz (1990:15) notes: „Wissenschaftliche Zeichnungen geben zwar vor, die Wirklichkeit und nichts als die Wirklichkeit abzubilden. Sie bilden jedoch die Wirklichkeit in Fragmenten unter bestimmten Gesichtspunkten heraus und entnehmen dem Vorbild des Gegenstandes jenes Zeichen, das zur Eigentlichkeit des Wirklichkeitsbefundes erklärt werden soll. “This way of looking at things may be true of illustrated identification keys which show taxonomically relevant characters in isolation and reduce them to the aspects important for differential diagnosis. However, drawings in the field of biology range across a broad spectrum from this kind of very abstract representation to naturalistic depictions which do not typologize but are based on real-life models. Naturalistic depictions include those designed to show the overall appearance of an animal as a whole, those designed to document mutants and developmental deformities, drawings of anatomical preparations, and drawings of the patterns left by certain wood pest infestations.

24 For a discussion of one of the few attempts to reconstruct illustrations to Aristotle’s zoological texts carried out to date see Fürst von Lieven and Humar (2017b:111‒113).
